# Metabolism and Pharmacokinetics of San-Huang-Xie-Xin-Tang, a Polyphenol-Rich Chinese Medicine Formula, in Rats and *Ex-Vivo* Antioxidant Activity

**DOI:** 10.1093/ecam/nep124

**Published:** 2011-06-07

**Authors:** Chi-Sheng Shia, Yu-Chi Hou, Shin-Hun Juang, Shang-Yuan Tsai, Pei-Hsun Hsieh, Lu-Ching Ho, Pei-Dawn Lee Chao

**Affiliations:** ^1^Institute of Pharmaceutical Chemistry, Taiwan; ^2^School of Pharmacy, China Medical University, 91 Hsueh-Shih Road, Taichung, Taiwan 404, ROC, Taiwan; ^3^Institute of Chinese Pharmaceutical Sciences, China Medical University, Taichung, ROC, Taiwan

## Abstract

San-Huang-Xie-Xin-Tang (SHXXT), a widely used Chinese herbal formula, consists of rhizomes of *Rheum officinale*, roots of *Scutellaria baicalensis* and rhizomes of *Coptis chinesis*. This study investigated the metabolism and pharmacokinetics of polyphenols in SHXXT, including baicalin, baicalein, wogonin, emodin, aloe-emodin, rhein and chrysophanol. The quantitation methods of SHXXT decoction and rat serum using high performance liquid chromatography were developed and validated in this study. After oral administration of SHXXT decoction to rats, the parent forms of various constituents and their conjugated metabolites in serum were determined before and after hydrolysis with **β**-glucuronidase and sulfatase. The results showed that only free form of rhein can be quantitated, whereas the parent forms of coptisine, palmatine, berberine, baicalein, wogonin, emodin, aloe-emodin and chrysophanol were not detected in serum. The glucuronides of baicalein, wogonin, emodin, aloe-emodin, rhein and chrysophanol were the predominant forms in bloodstream. In order to evaluate the *in vivo* antioxidant activity of SHXXT, the serum metabolite of SHXXT was prepared, characterized and followed by evaluation of the effect on AAPH-induced hemolysis. The results indicated that metabolites of SHXXT exhibited significant free radical scavenging activity. We suggest that biologists redirect their focus to the bioactivity of the conjugated metabolites of these polyphenols.

## 1. Introduction

**Figure 1:**
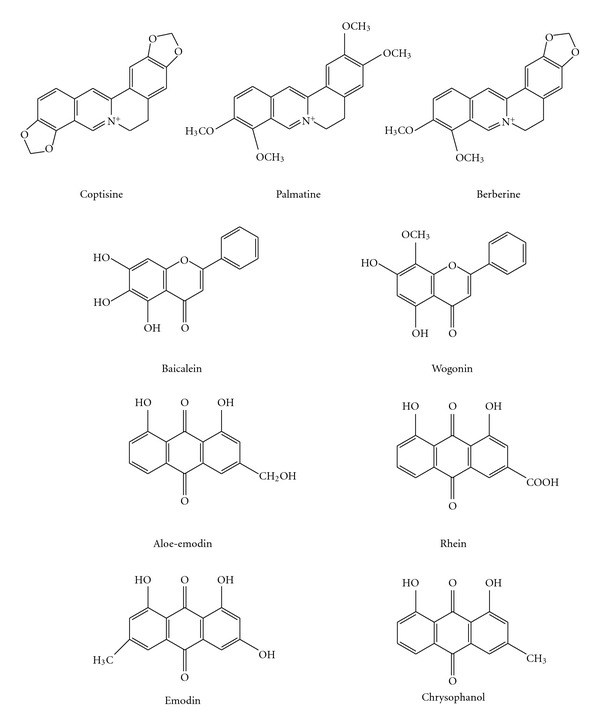
Structures of alkaloids and polyphenols in SHXXT.

San-Huang-Xie-Xin-Tang (SHXXT) is a popular Chinese medicine formula, containing Rhei Rhizoma (rhizomes of *Rheum officinale* BAILL; RR), Scutellariae Radix (roots of *Scutellaria baicalensis* Georgi; SR) and Coptidis Rhizoma (rhizomes of *Coptis chinesis* Franch; CR). Numerous studies have reported bioactivities of SHXXT including hypotension [1, 2], gastric protection [3, 4], antioxidant [5] and anti-inflammatory effects [6–8]. The chemical composition of SHXXT is very complex and the major constituents known are anthraquinones in RR including emodin, aloe-emodin, rhein, chrysophanol and their glycosides; flavonoids in SR including baicalin, baicalein, wogonoside, wogonin and alkaloids in CR including berberine, palmatine, coptisine (structures shown in Figure 1). However, the information concerning biological fates of various constituents in SHXXT remains lacking, which prevents us from better understanding the rational of its clinical implication.

On the basis of recent findings on the metabolism of flavonoid polyphenols, it is increasingly recognized that polyphenol glycosides are subject to hydrolysis in gut lumen, absorbed as their aglycones and then extensively metabolized by conjugation reactions [9]. This study investigated the metabolism and pharmacokinetics of polyphenolic derivatives including anthraquinones, flavonoids and isoquinoline alkaloids after administration of SHXXT decoction to rats.

For more than two decades, the free radical-mediated peroxidation of membrane lipid and oxidative damage of DNA has long been thought to be associated with a variety of health problems, such as cancer, atherosclerosis, neurodegenerative diseases and aging. As an extension of our pharmacokinetic study, the serum metabolite of SHXXT in rats was prepared, characterized and the activity against 2,2′-azobis(2-amidinopropane hydrochloride) (AAPH)-induced hemolysis was evaluated.

## 2. Methods

### 2.1. Materials and Reagents

RR, SR and CR were purchased from a Chinese drugstore in Taichung. The origin of the crude drugs were identified by microscopic examination by one of the authors (Y.-C.H.). Voucher specimens were deposited in China Medical University. Baicalein (purity 98%), and wogonin (purity 98%) were supplied by Wako (Osaka, Japan). Aloe-emodin (purity 95%), rhein (purity 95%), emodin (purity 90%), chrysophanol (purity 98%), berberine (purity 99%), palmatine (purity 97%), coptisine (purity 98%), *β*-glucosidase, *β*-glucuronidase (type B-1 from bovine liver), sulfatase (type H-1 from *Helix pomatia*, containing 14 000 units g^−1^ of sulfatase and 498 800 units g^−1^ of *β*-glucuronidase) and 2-methlylanthraquinone (purity 95%) were purchased from Sigma Chemical Co. (St Louis, MO, USA).

### 2.2. Preparation of SHXXT Decoction

Crude drugs of RR, SR and CR (2 : 1 : 1) were weighed and boiled in 20-fold volume of water and heating was carried out on a gas stove. After boiling, gentle heating continued until the volume reduced to <10-fold volume. The mixture was filtered with gauze while hot and sufficient water was added to make 10-fold volume and frozen at −30°C for later use.

### 2.3. Quantitation of Alkaloids, Polyphenols and Related Glycosides in SHXXT Decoction

SHXXT decoction (3.0 ml) was mixed with 7.0 ml of methanol and centrifuged. The properly diluted supernatant (200 *μ*l) was added with 200 *μ*l of 2-methylanthraquinone solution (10.0 *μ*g ml^−1^ in methanol) as internal standard and 20 *μ*l were subjected to high performance liquid chromatography (HPLC) analysis. The HPLC apparatus included a pump (LC-10AT, Shimadzu, Kyoto, Japan), an UV detector (SPD-10AVP, Shimadzu) and an automatic injector (Series 200 Autosampler, Perkin Elmer, USA). The Cosmosil 5C18-ARII column (4.6 × 250 mm, 5 *μ*m) was equipped with a guard column (4.6 × 50 mm, 5 *μ*m) (GL Science Inc., Tokyo, Japan). The mobile phase consisted of acetonitrile (A) –0.1% phosphoric acid (B) and programmed as follows: A/B: 23/77 (0–10 min), 20/80 (15–25 min), 22/78 (30–45 min), 38/62 (50–60 min), 70/30 (65–95 min) and 23/77 (100–105 min). The flow rate was programmed as follows: 1.0 ml min^−1^ (0–68 min), 0.2 ml min^−1^ (68–78 min), 1.0 ml min^−1^ (78–100 min). The detection wavelength was set at 250 nm.

Hydrolysis of 1 ml of SHXXT decoction was conducted by incubating with 1 ml of 50 units ml^−1^ of *β*-glucosidase solution (in acetate buffer, pH 5) at 37°C for 3 h, followed by the addition of sufficient water to make 2.0 ml. Then 2.0 ml of internal standard solution (10.0 *μ*g ml^−1^ 2-methylanthraquinone in methanol) was added and 20 *μ*l of the mixture was subjected to HPLC analysis. The glycoside contents of baicalein, wogonin, aloe-emodin, rhein, emodin and chrysophanol were calculated by subtracting the concentrations of each aglycone in the decoction from those of correspondent aglycone in the hydrolysate.

### 2.4. Metabolism and Pharmacokinetics of SHXXT in Rats

#### 2.4.1. Animals and Drug Administration

Male Sprague-Dawley rats were supplied by National Laboratory Animal Center (Taipei, Taiwan) and housed in a 12-h light–dark cycle, constant temperature environment prior to study at the Animal Center of China Medical University (Taichung, Taiwan). Twelve male Sprague-Dawley rats weighing 320–450 g were fasted for 12 hours before drug administration and food was withheld for another 3 h. Rats were orally given 10 ml kg^−1^ of SHXXT decoction, equivalent to 5 g kg^−1^ of crude drugs. Water was supplied *ad libitum*.

#### 2.4.2. Blood Sample Collection

Blood samples (1.0 ml) were withdrawn via cardiac puncture before and at 10, 30, 60, 240, 480, 720, 1440 and 2880 min after administration of SHXXT decoction. Blood samples were centrifuged to obtain serum, which was stored at −30°C for later analysis. This animal study adhered to *The Guidebook for the Care and Use of Laboratory Animals (2002)* (Published by The Chinese Society for the Laboratory Animal Science, Taiwan, ROC). The Committee of Animal Management in China Medical University approved the animal study.

#### 2.4.3. Quantitation of Polyphenols and Their Conjugated Metabolites in Serum

The conjugated metabolites in serum were determined through hydrolysis with glucuronidase and sulfatase. Serum (150 *μ*l) was mixed with 150 *μ*l of *β*-glucuronidase (2000 units ml^−1^ in pH 5 acetate buffer) or sulfatase (1000 units ml^−1^ containing 35 600 units ml^−1^ of *β*-glucuronidase in pH 5 acetate buffer), 50 *μ*l of ascorbic acid (150 mg ml^−1^) and incubated at 37°C for 4 hours. After hydrolysis, serum was added with 50 *μ*l of 0.1 N HCl and partitioned with 400 *μ*l of ethyl acetate (containing 0.125 *μ*g ml^−1^ of 2-methylanthraquinone as the internal standard) and then centrifuged at 10 000 g for 15 min. The ethyl acetate layer was evaporated under nitrogen to dryness and reconstituted with mobile phase for HPLC analysis.

For the determination of free forms of polyphenols, serum (150 *μ*l) was added with 50 *μ*l of 0.1 N HCl, 150 *μ*l of pH 5 acetate buffer, 50 *μ*l of ascorbic acid (150 mg ml^−1^) and partitioned with 400 *μ*l of ethyl acetate (containing 0.125 *μ*g ml^−1^ of 2-methylanthraquinone as the internal standard). The ethyl acetate layer was concentrated under nitrogen and reconstituted with mobile phase, then subject to HPLC analysis. On the other hand, gradient elution using mixture of acetonitrile (A) and 0.1% phosphoric acid (B) as the mobile phase was programmed as follows: A/B: 30/70 (0 min); 70/30 (25 min), 80/20 (26–30 min) and 30/70 (40–45 min). The detection wavelength was set at 250 nm and the flow rate was 0.8 ml min^−1^. The serum standards of baicalein, aloe-emodin, wogonin, rhein, emodin, chrysophanol were in the concentration ranges of 0.3–20.0, 0.2–10.0, 0.2–5.0, 0.2–10.0, 0.2–10.0 and 0.2–5.0 *μ*g ml^−1^, respectively.

#### 2.4.4. Validation of the Assay Methods

The system suitability was evaluated through analysis of precision and accuracy. The precision was evaluated using intra-day and inter-day assays of standards three times daily and over three consecutive days. The accuracy of the system was expressed by the relative error of the mean calculated concentration to the real concentration of each calibrator. The recoveries of each compound from serum were determined by comparing the peak area of extracted serum standards to the peak area of unextracted standards spiked in extracted serum. The LLOQ (lower limit of quantitation) represents the lowest concentration of analysis in a sample that can be determined with acceptable precision and accuracy, whereas LOD (limit of detection) represents the lowest concentration of analysis in a sample that can be detected (with signal/noise >3).

### 2.5. Antioxidant Activity of Serum Metabolites of SHXXT

#### 2.5.1. Preparation of Erythrocytes Suspension

Four rats were fasted for 12 hours, blood was withdrawn via cardiac puncture and then collected into vacutainer tubes containing EDTA. After removing plasma and buffy coat, erythrocytes were washed five times with two volumes of cold phosphate-buffered saline (PBS). During the last wash, the erythrocytes were centrifuged at 2500 g for 10 min to obtain a packed cell preparation. The packed erythrocytes were then suspended in four volumes of PBS solution.

#### 2.5.2. Preparation and Characterization of Serum Metabolites of SHXXT

After overnight fast, five Sprague-Dawley rats were administered orally with 5.0 g kg^−1^ of SHXXT decoction via gastric gavage. Half an hour later, a second dose was boosted. At 30 min after the second dose, blood was withdrawn from rats to obtain serum. Four volumes of methanol was mixed with serum and centrifuged to remove proteins. The supernatant was evaporated under vacuum to dryness and the residue was dissolved with water. The aqueous solutions of metabolites were lyophilized to obtain powders and stored at −80°C, of which an aliquot was quantitated following the procedures described earlier for serum assay.

#### 2.5.3. AAPH-induced Hemolysis Assay

The serum metabolite of SHXXT was reconstituted with PBS to afford 1-, 1/2- and 1/8-fold of serum levels. Besides, blank serum was collected from rats after overnight fast and processed in the same manner to prepare a sample of blank serum as control. To 100 *μ*l of erythrocyte suspension, the mixtures of 100 *μ*l of 200 mM AAPH (in PBS) and 200 *μ*l of PBS containing various concentrations of SHXXT serum metabolites were added. The reaction mixture was shaken gently and incubated at 37°C for 0, 1, 2, 3, 4 and 5 hours. After incubation, the reaction mixture was added with 600 *μ*l of PBS and centrifuged at 10 000 g for 1 min. The percentage of hemolysis was determined by measuring the absorbance at 540 nm and compared with that of complete hemolysis.

### 2.6. Data Analysis

The peak serum concentration (*C*_max_) was recorded as observed. Noncompartment model of WINNONLIN (version 1.1, SCI software, Statistical Consulting Inc., Apex, NC, USA) was used for the computation of pharmacokinetic parameters. The area under the serum concentration-time curve (AUC_0−*t*_) was calculated using trapezoidal rule to the last point. Data for the percentage of hemolysis of among groups were statistically compared using ANOVA followed by Scheffe's *post hoc* test. A level of probability of ≤0.05 was considered to be significant.

## 3. Results

### 3.1. Quantitation of Alkaloids, Polyphenols and Related Glycosides in SHXXT Decoction

**Figure 2:**
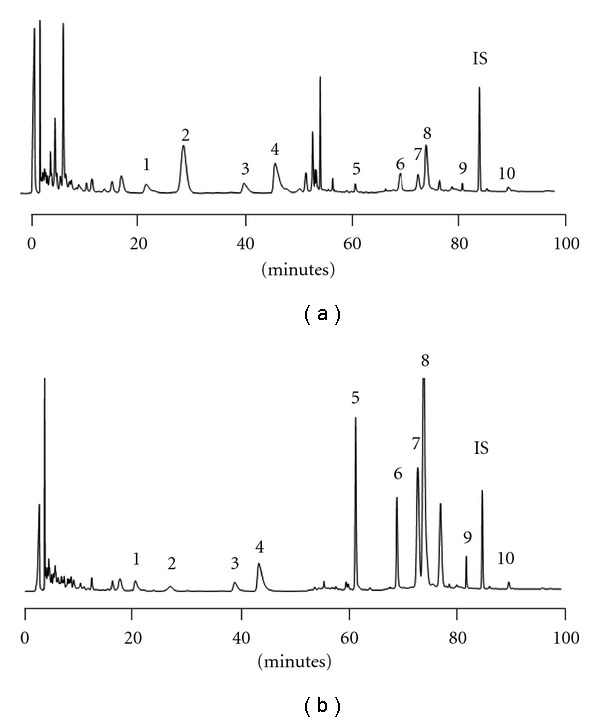
HPLC chromatograms of SHXXT decoction (a) and its hydrolysate (b) 1: coptisine, 2: baicalin, 3: palmatine, 4: berberine, 5: baicalein, 6: aloe-emodin, 7: wogonin, 8: rhein, 9: emodin, 10: chrysophanol, IS: 2-methylanthraquinone.

Figure 2(a) shows the HPLC chromatogram of SHXXT decoction. Good linear relationships were obtained in the concentration ranges of 3.1–100.0, 3.1–100.0, 15.6–500.0, 12.5–400.0, 7.8–250.0, 0.8–25.0, 3.1–100.0, 3.1–100.0, 0.3–10.0 and 0.3–10.0 *μ*g ml^−1^ for coptisine, palmatin, berberine, baicalin, baicalein, aloe-emodin, wogonin, rhein, emodin and chrysophanol, respectively. Validation of the method indicated that the coefficients of variation were <10% and the relative errors were <20% for intra-day and inter-day analysis. Hydrolysis of SHXXT decoction using *β*-glucosidase resulted the chromatogram shown in Figure 2(b), indicating that the polyphenol peaks were markedly increased. The contents of various constituents with related glycosides in the decoction were listed in Table 1. The relative abundance of each constituent (aglycone + glycosides) was as follows: baicalein > berberine > rhein > wogonin > coptisine > palmatine, aloe-emodin > emodin > chrysophanol. 

### 3.2. Metabolism and Pharmacokinetics of SHXXT in Rats

**Figure 3:**
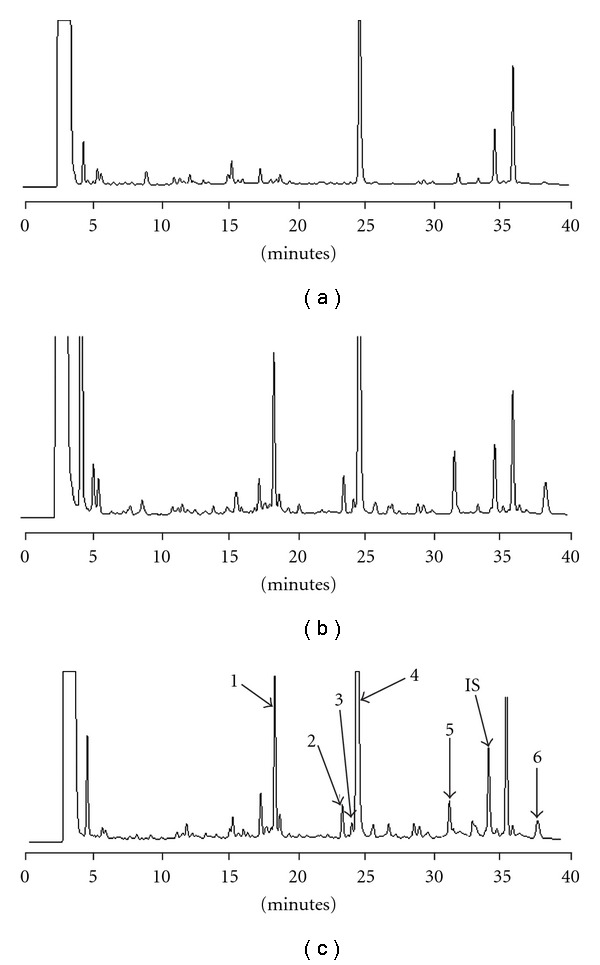
HPLC chromatograms of rat serum after administration of SHXXT. (a) serum specimen before hydrolysis (b) serum specimen hydrolyzed with *β*-glucuronidase (c) serum specimen hydrolyzed with sulfatase/glucuronidase 1: baicalein, 2: aloe-emodin, 3: wogonin, 4: rhein, 5: emodin, 6: chrysophanol, IS: 2-methylanthraquinone.

Our preliminary study using 4-fold methanol to deproteinize the serum revealed the absence of berberine, palmatine and coptisine. Typical HPLC chromatograms of serum sample before and after treatments with glucuronidase and sulfatase are shown in Figure 3, indicating that besides rhein, the parent forms of baicalein, wogonin, emodin, aloe-emodin and chrysophanol were not present in serum. However, after treatments with glucuronidase and sulfatase, the peaks of baicalein, wogonin, emodin, aloe-emodin and chrysophanol emerged and the peak of rhein was significantly enhanced, a clear indication that the major molecules in the bloodstream were their conjugated metabolites. Good linearities were shown in the ranges of 0.3–20.0 *μ*g ml^−1^ for baicalein, 0.2–5.0 *μ*g ml^−1^ for wogonin, 0.2–10.0 *μ*g ml^−1^ for emodin, aloe-emodin and rhein and 0.1–5.0 *μ*g ml^−1^ for chrysophanol in serum. Validation of the method indicated that the coefficients of variation were less than 10% and the relative errors were <20% for intra-day and inter-day analysis. The recoveries of each compound from serum were satisfactory.

**Figure 4:**
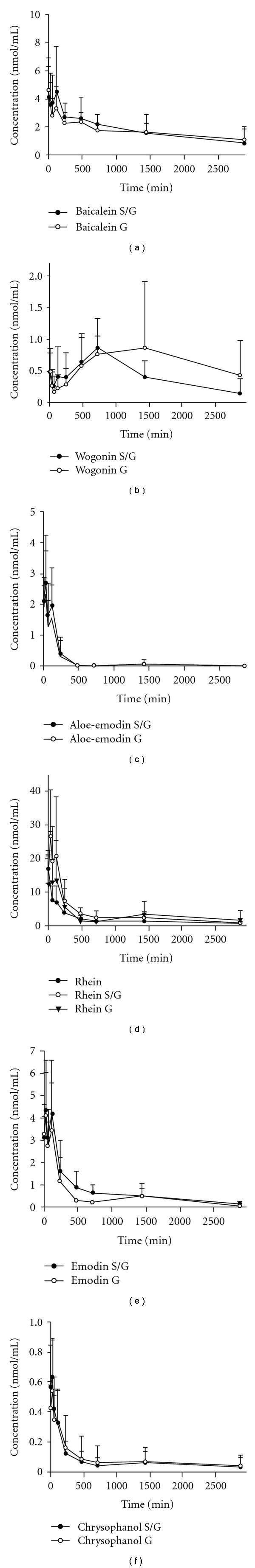
Mean (±SE) serum concentration–time profiles of sulfates/glucuronides (S/G), glucuronides (G) of various constituents and free form of rhein after oral administration of SHXXT decoction in nine rats.

Figure 4 depicts the mean serum concentration-time profiles of various constituents and their conjugated metabolites in rats after administration of SHXXT. The pharmacokinetic parameters are listed in Table 2. Of flavonoids, the *C*_max_ and AUC_0−*t*_ of baicalein glucuronides/sulfates were higher than those of wogonin glucuronides/sulfates. Among anthraquinones, the *C*_max_ and AUC_0−*t*_ of rhein and its sulfates/glucuronides were higher than others, whereas those of chrysophanol sulfates/glucuronides were the lowest. The relative systemic exposure of each polyphenol with their conjugated metabolites was ranked as follows: rhein > baicalein > emodin > wogonin > aloe-emodin > chrysophanol. The residence times of the conjugated metabolites of various polyphenols were quite long except aloe-emodin.

### 3.3. Inhibition of Serum Metabolites of SHXXT on AAPH-Induced Hemolysis

**Figure 5:**
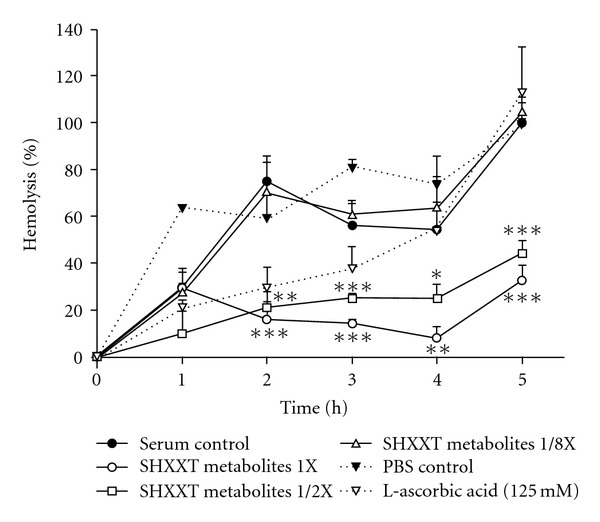
Effects of various serum levels of SHXXT metabolites on APPH-induced hemolysis. Data are expressed as mean ± SD of triplicates. *P < .05, **P < .01, ***P < .001.

The serum metabolites of SHXXT used for measuring antioxidant activity have been characterized and the result is shown in Table 3. During incubation with erythrocytes and AAPH for 5 hours, the effects of 1-, 1/2- and 1/8-fold of SHXXT blood concentrations against hemolysis are shown in Figure 5. The serum metabolites of SHXXT at 1- and 1/2-fold of blood level exhibited significant free radical scavenging effect, whereas 1/8-fold was ineffective. 

## 4. Discussion

Polyphenols are predominantly present in plants as glycosides. Because authentic compounds of polyphenol glycosides were mostly not available, hydrolysis of SHXXT was then performed in order to quantitate the total content of each polyphenol with correspondent glycosides. When hydrolysis was carried out in 1.2N HCl, serious charring was observed. Alternatively, *β*-glucosidase was used for the hydrolysis and conducted at 37°C [10]. The analytical methods of SHXXT decoction and serum were developed in this study and validation of these methods indicated that the precision and accuracy were satisfactory.

Following oral administration of SHXXT, only rhein existed in part as free form, whereas the parent forms of berberine, palmatine, coptisine, baicalein, wogonin, aloe-emodin, emodin and chrysophanol were not found. The serum level of rhein, an anthraquinone carboxylic acid, was rather high, which can be accounted for by the low glucuronidation activity of UDP-glucuronyltransferases toward the class of carboxylic acids [11]. The absence of berberine, palmatine and coptisine in the blood can be explained by extensive first pass effect on account of that several metabolites of berberine have been detected in human urine and rat plasma after intake of berberine [12, 13]. The major metabolites identified in human urine included jatrorrhizine-3-sulfate, thalifendine-2-sulfate, demethyleneberberine-10-sulfate and berberrubine [12]. In rat plasma, the free forms and glucuronides of thalifendine, demethyleneberberine and jatrorrhizine were identified [13]. These metabolites of berberine were formed through dealkylation or/and conjugation reaction occurring in gut and liver during the first pass. Being salt-like compounds, berberine, palmatine and coptisine are seemingly too hydrophilic to be absorbed through passive diffusion. Lately, the absorption of berberine was found mediated by organic cationic transporter [14].

In regard to baicalein, wogonin, aloe-emodin, emodin and chrysophanol, only their conjugated metabolites were found in serum, indicating that they were subject to extensive conjugation metabolism by intestine and liver during the first pass. Since the authentic compounds of the conjugated metabolites of various polyphenols were not available, their concentrations in serum were quantitated indirectly through hydrolysis with glucuronidase and sulfatase. The hydrolysis condition has been optimized in our preliminary study. The optimal durations needed for treatments with glucuronidase and sulfatase were both 4 hours in the presence of ascorbic acid and under anaerobic condition. The addition of ascorbic acid was to avoid the oxidative decay of polypenol aglycones during the enzymolysis reaction.

**Figure 6:**
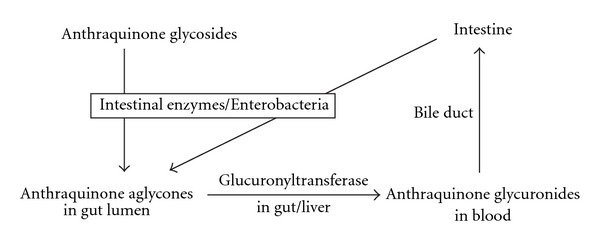
Biological fates of anthraquinone polyphenols in rats.

Due to considerable amount of glucuronidase in the sulfatase (type H-1) used in this study, treatment with this enzyme resulted in the hydrolysis of both sulfates and glucuronides. The results showed that the serum profiles of baicalein, wogonin, rhein, aloe-emodin, emodin and chrysophanol liberated by glucuronidase and sulfatase/glucuronidase were comparable, indicating that the glucuronides were the principal metabolites, whereas their sulfates were negligible. The mean residence times of the glucuronides of various polyphenols were rather long, indicating possible enterohepatic recycling of these metabolites. Because the biotransformations of flavonoids *in vivo* have been generally known, the biological fates of anthraquinone polyphenols in rats is proposed in Figure 6 based on our results.

In the wake of getting the ratios of total AUC_0−*t*_ (glucuronides/sulfates + free form) to dose (aglycones + glycosides) and compared among six polyphenols (data not shown), the relative bioavailability of polyphenols can be ranked as follows: rhein > emodin > baicalein, chrysophanol, wogonin > aloe-emodin. The fact that rhein shows profoundly higher bioavailability than other polyphenols can be in part accounted for by the underestimated dose, because rhein can be biotransformed from aloe-emodin [15] and bianthrones such as sennosides A and B [16], which had not been quantitated in this study. In an *in vitro* study, we did find that considerable amount of rhein emerged at once when sennosides A and B were incubated with feces of rats and rabbits (data not shown). On the other hand, aloe-emodin was found the least bioavailable, which can be explained by its poor solubility in various solvent and its *in vivo* conversion to rhein [15].

In the AAPH-induced hemolysis assay, our results suggested that the metabolite of SHXXT exhibited promising free radical scavenging activity compared to blank serum. The potential protection of erythrocyte membrane from free-radical attack provides an important pathophysiological basis for making use of SHXXT as a remedy for free-radical related diseases such as cancer, atherosclerosis, neurodegenerative diseases and aging.

Despite voluminous *in vitro* bioactivity studies reporting various beneficial effects of polyphenols [17–21], our finding that virtual absence of the free forms of baicalein, wogonin, aloe-emodin, emodin and chrysophanol suggests that it is difficult to infer the *in vivo* effects of these compounds from their *in vitro* activities. In fact, the principle metabolites *in vivo* were their glucuronides, which possess completely different physicochemical properties from their free forms. These metabolites should play more important role for *in vivo* activities than their parent forms. It is an important issue that biologists redirect their targets on the conjugated metabolites of polyphenols. Several recent studies actually found the sulfates/glucuronides of morin and quercetin showed more promising bioactivities than their free forms [22–24], pointing to the possibility that the conjugated metabolites of polyphenols were not necessarily inactive and might be the principal active forms.

## 5. Conclusion

In SHXXT, alkaloids including berberine, palmatine, coptisine and polyphenols except rhein were not absorbed *per se*. Glucuronides were the principal metabolites of polyphenols including baicalein, wogonin, rhein, aloe-emodin, emodin and chrysophanol. For better understanding the rational of the clinical implications of polyphenol-rich botanical products, it is strongly advised that biologists pay more attention to the bioactivity and toxicity of the metabolites of these polyphenols.

## Funding

National Science Council, ROC (NSC95-2320-B-039-023-MY2, NSC 96-2320-B-039-037-MY3), the Committee on Chinese Medicine and Pharmacy, ROC (CCMP96-RD-019, CCMP94-RD-019) and China Medical University, Taichung, Taiwan, ROC (CMU95-087).

## Figures and Tables

**Table 1:** Contents (mmol) of various constituents in the hydrolysate of SHXXT (5 g) decoction.

Constituents	Contents
Coptisine	21.4
Palmatine	16.6
Berberine	60.0
Baicalein	118.8
Aloe-emodin	16.5
Wogonin	32.8
Rhein	49.5
Emodin	9.8
Chrysophanol	2.0

**Table 2:** Pharmacokinetic parameters of various constituents and their sulfates/glucuronides (S/G) and glucuronides (G) after oral administration of SHXXT (5 g kg^−1^) to nine rats.

Constituents	Metabolites	*C*_max_ (nmol ml^−1^)	AUC_0−2880_ (nmol min ml^−1^)	MRT_0−2880_ (min)
Baicalein	S/G	5.7 ± 2.8	4733.9 ± 1850.2	920.8 ± 331.9
	G	5.0 ± 2.2	4553.1 ± 2062.2	1081.0 ± 542.2
Wogonin	S/G	1.1 ± 0.4	1045.5 ± 491.1	960.7 ± 296.3
	G	1.3 ± 0.8	1479.8 ± 1142.7	1165.0 ± 403.9
Aloe-emodin	S/G	3.0 ± 1.3	340.4 ± 176.1	104.4 ± 65.7
	G	2.7 ± 1.4	294.6 ± 165.4	207.8 ± 216.0
Rhein	Free form	17.5 ± 4.6	5249.5 ± 3838.8	692.0 ± 293.9
	S/G	27.8 ± 15.2	9348.9 ± 5510.3	632.0 ± 226.6
	G	22.7 ± 9.0	8736.1 ± 5299.0	779.8 ± 616.4
Emodin	S/G	5.0 ± 2.2	1929.4 ± 1012.6	570.9 ± 198.0
	G	4.6 ± 2.0	1221.8 ± 379.3	503.3 ± 388.1
Chrysophanol	S/G	0.7 ± 0.3	169.1 ± 79.6	629.4 ± 502.5
	G	0.6 ± 0.3	221.1 ± 209.5	624.3 ± 604.1

**Table 3:** Contents (*μ*mol ml^−1^) of various constituents in 1-fold serum level of SHXXT metabolite used for anti-hemolysis test.

	Baicalein	Aloe- emodin	Rhein	Emodin	Chrysophanol
Free form	ND	ND	2.1	ND	ND
Sulfates/ glucuronides	1.8	1.3	7.9	0.7	3.9
Glucuronides	1.1	0.2	1.5	0.5	1.3

ND: not detected.
